# A hierarchical Bayesian approach to multiple testing in disease mapping

**DOI:** 10.1002/bimj.200900209

**Published:** 2010-12

**Authors:** Dolores Catelan, Corrado Lagazio, Annibale Biggeri

**Affiliations:** Department of Statistics “G.Parenti”, University of Florenceviale Morgagni, 59, 50134 Florence, Italy; Biostatistics Unit, ISPO Cancer Prevention and Research Institutevia Cosimo il Vecchio, 2, 50139 Florence, Italy; Department of Statistical Sciences, University of Udinevia Treppo 18, 33100 Udine, Italy

**Keywords:** Disease mapping, False discovery rate, Hierarchical Bayesian models, Multiple testing, Posterior probabilities

## Abstract

We propose a Bayesian approach to multiple testing in disease mapping. This study was motivated by a real example regarding the mortality rate for lung cancer, males, in the Tuscan region (Italy). The data are relative to the period 1995–1999 for 287 municipalities. We develop a tri-level hierarchical Bayesian model to estimate for each area the posterior classification probability that is the posterior probability that the municipality belongs to the set of non-divergent areas. We show also the connections of our model with the false discovery rate approach. Posterior classification probabilities are used to explore areas at divergent risk from the reference while controlling for multiple testing. We consider both the Poisson-Gamma and the Besag, York and Mollié model to account for extra Poisson variability in our Bayesian formulation. Posterior inference on classification probabilities is highly dependent on the choice of the prior. We perform a sensitivity analysis and suggest how to rely on subject-specific information to derive informative *a priori* distributions. Hierarchical Bayesian models provide a sensible way to model classification probabilities in the context of disease mapping.

## 1 Introduction

Generally speaking, epidemiological surveillance consists of continuously gathering and analyzing data for changes in disease occurrence (Last, 2001). Surveillance may be based on time or space or a combination of time-space, with an active or passive approach. Disease mapping, *i.e.* the study of the variability of disease occurrence on space, is a cornerstone of epidemiologic surveillance. Currently, the availability of data on a small scale makes it popular to scan for abnormal disease rates potentially associated with widespread environmental exposures or to search for a localized cluster of cases in proximity of putative sources of pollution (Elliott *et al.*, 2000). In disease mapping, a moderate to large number of area-level relative risks are considered. However, the large heterogeneity of population density among small areas leads to smaller *p*-values paradoxically associated with relative risk estimates closer to the null. Such inconsistency justified the development of shrinkage estimators (Clayton and Kaldor, 1987). Shrinkage estimators, as empirical Bayes or full Bayes, are now accepted as standard tools in spatial epidemiology, but they leave unresolved the multiple comparison problem.

Control of Family Wise Error Rate (FWER) that is a global control of type I error is generally pursued in the Surveillance framework (Frisén, 2003; Kulldorff, 2001). In his article of 2007, Rolka discussed the cost in sensitivity of adopting a FWER control procedure and he mentioned control of the False Discovery Rate (FDR). FDR is the rate of false positives among all rejected hypotheses and was introduced with examples in the context of clinical trials by Benjamini and Hochberg (1995). FDR has a Bayesian interpretation and it is connected to the *q*-value, a Bayesian alternative to the *p*-value (Storey, 2003).

In the disease mapping literature, posterior probability for each area having a risk higher than a predefined threshold after having specified an appropriate hierarchical Bayesian model, was suggested as a way to screen areas at higher/lower risk (Bernardinelli and Montomoli, 1992; Richardson *et al.*, 2004). This is not sufficient to assure that the posterior inference adjusts for multiple testing. To accomplish this task, the probability model needs to include a null prior and related hyperparameters that define the prior probability mass for non-*divergent* areas (Scott and Berger, 2006). In the following article, we consider a two-sided alternative hypothesis and use the term *divergent* to denote areas at risk different from the null. This meaning of the word divergent was used by Olhssen *et al.* (2007).

### 1.1 Aim of the study

This article aims to develop a hierarchical Bayesian modeling approach to multiple testing in the context of disease mapping. The idea to use an FDR approach instead of an FWER control is based upon the fact that the erroneous rejection of the null hypothesis for some municipalities does not challenge the result of the whole descriptive analysis whose aim is to assess heterogeneity of risk in the entire study region. Therefore, the FWER control is too strict for the application's needs (Benjamini, 2009).

In the following analysis a tri-level hierarchical Bayesian model is proposed to estimate for each area the probability of belonging to the null, to be used to explore areas at divergent risk (higher or lower then the reference disease rate) while controlling for multiple testing. We took advantage of real data regarding the mortality rate due to lung cancer in males at the municipal level in the Tuscan region (Italy) during the period 1995–1999.

In Section 2, we describe the mortality data. In Section 3, we briefly introduce the problem of multiple comparisons; we then describe the proposed hierarchical Bayesian models for disease mapping and how to estimate posterior classification probabilities. The results are presented in Section 4. The conclusion and discussion follow in Section 5.

## 2 Motivating example

Lung cancer death certificates, for the period 1995–1999, were considered for male residents in the 287 municipalities of the Tuscan region (Italy). Data were made available by the Regional Mortality Register. The expected number of cases for each municipality was computed under indirect standardization applying a set of age-specific (18 age classes, 0–4,…, 85 or more) reference rates (Tuscany, 1971–1999) to the population of each area.

The task is to identify municipalities with a divergent risk from the reference (two-sided alternative). In fact for each *i*-th area out of 287, the standardized mortality ratio (observed/expected number of cases) is an estimate of the relative risk (RR), *i.e.* the disease risk in each area compared with the adopted standard. We have an implicit identical null hypothesis *H*_0_: RR_*i*_=1 for each area, because of the indirect standardization (Breslow and Day, 1987).

## 3 Statistical modeling

### 3.1 Multiple comparisons: general aspects

Let *m* be the number of hypothesis tests. The commonly controlled quantity to account for multiple testing is the FWER, and the most common method is the Bonferroni approach that is if we fix the type I error probability to α and *m* hypothesis tests are performed, then each test is controlled at a level of α/*m* (Bonferroni, 1936). This guarantees that the probability of at least one false positive is at max equal to α.

As the *m* null hypotheses have different implications, it can be argued that the FWER approach is too strict. Benjamini and Hochberg (1995) proposed a way to control the proportion of false rejections among the total number of rejections and introduced the FDR.

In particular, let *I*_*i*_ define an indicator for rejecting *H*_0_ for the *i*-th area, and *R*=∑*I*_*i*_ as the total number of rejections. Define also *r*_*i*_∈{0, 1} the indicator of the unknown true status, *i.e.* the indicator that the *i*-th area is truly not divergent from the reference.

The False Discovery Proportion (FDP) ∑(*r*_*i*_×*I*_*i*_)/*R* is the fraction of false rejections over the total number of rejections (Genovese and Wasserman, 2006). Benjamini and Hochberg (1995) consider controlling the expected value of FDP, taking the expectation over repeated experiments. Let *V* define the number of false rejections over *m* hypothesis tests. The FDR is the expected value *E*(*V*/*R*), which can be factored as *E*[(*V*/*R*)∣*R*>0]Prob(*R*>0) (Benjamini and Hochberg, 1995). Storey (2003) concentrated on *E*[(*V*/*R*)∣*R*>0], a piece of the FDR, which is known as the positive FDR (pFDR).

The pFDR is defined only when at least one rejection occurred (one positive result). We cannot control pFDR because if all null hypotheses are true, any rejection is false (Storey, 2003). The pFDR can be interpreted as a posterior Bayesian probability and can be used to define the *q*-value, Prob(*H*_0_ ∣ *Y*≥*Y*_obs_), *Y* being a test statistic and *Y*_obs_ the observed value, for a generic *i*-th test. The *q*-value is the minimum pFDR that can occur rejecting the null hypothesis on the basis of the observed or more extreme values of the test statistic. The *q*-value is a measure that takes into account multiple testing and represents how far we are from the null hypothesis on the basis of observed data. The “frequentist” estimate of the *q*-value is reported in Storey (2003). It is based on the ordered *p*-values and it uses information on the proportion of true null hypotheses. As a referee pointed out, the frequentist estimate of the *q*-value reduces to the adaptive control of the Benjamini–Hochberg procedure (Benjamini and Hochberg, 2000; Benjamini *et al.*, 2006).

Efron (2005, 2007, http://www-stat.stanford.edu/~ckirby/brad/papers/2005LocalFDR.pdf) focused on Prob(*H*_0_ ∣ *Y*=*Y*_*i*_; **Y**, **ξ)**=Prob(*r*_*i*_=1 ∣ *Y*=*Y*_*i*_; **Y**, **ξ**) the local FDR that is the posterior probability of the null hypothesis conditional of observed vector **Y** and hyperparameter vector **ξ**. Dealing with the local FDR differs from thresholding *q*-values. Generally speaking, the *q*-value averages over the values of the test statistic equal to or greater than *Y*_*i*_. As we apply tail probabilities the *q*-value will be less conservative than local FDR.

Efron's approach is empirical Bayes and the local FDR is calculated at some empirical value of ξ. A full Bayes approach would provide Prob(*H*_0_ ∣ *Y*=*Y*_*i*_; **Y**)=Prob(*r*_*i*_=1 ∣ *Y*=*Y*_i_; **Y**), the marginal posterior probabilities, having defined a density for **ξ** (Müller *et al.*, 2007).

In Section 3.2, we will briefly describe the bi-level hierarchical Bayesian models commonly used in the context of disease mapping to estimate area relative risks. In Section 3.3, we will extend these models to a tri-level Bayesian formulation to obtain fully Bayesian estimates of Prob(*r*_i_=1∣=*Y*_*i*_; *Y*), which we call posterior classification probabilities.

The reader should note that we use the term “classification probabilities” to underline the connection to the classification theory and clearly distinguish it from Prob(θ_*i*_>1∣**Y**), which are often denoted as posterior probabilities. Barbieri and Berger (2004), referring to the problem of covariate selection, called posterior inclusion probability Prob(*r*_*i*_=0 ∣ *Y*=*Y*_*i*_; **Y**)=1 - Prob(*r*_*i*_=1 ∣ *Y*=*Y*_*i*_; **Y**).

### 3.2 Hierarchical models for disease mapping

Disease mapping consists of a cartographical representation of the spatial distribution of a measure of disease risk. Disease atlases in the recent years have been produced at fine spatial resolution. Risk estimates from small areas suffer from substantial extra variability and several statistical approaches have been proposed to overcome such difficulties and “stabilize” risk estimates. All these approaches rely on shrinkage estimators and among these Bayesian estimators appear to be the most appealing (Lawson *et al.*, 1999; Elliott *et al.*, 2000; SMMR, 2005). In this section, we describe the two most common Bayesian hierarchical models used to smooth risk estimates in disease mapping: the Poisson-Gamma model (Clayton and Kaldor, 1987), and the Besag-York-Mollié (here after known as BYM) model (Besag *et al.*, 1991). These models will be used within our tri-level hierarchical Bayesian model in the following section.

Let area counts of disease *Y*_*i*_ (*i*=1, …, 287) follow a Poisson distribution with mean *E*_*i*_×θ_*i*_, where *E*_*i*_ is the expected number of cases under indirect standardization and θ_*i*_, is the relative risk. The maximum likelihood estimator of θ_*i*_ is called the standardized mortality ratio (SMR: 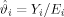).

The precision of the SMR is proportional to *E*_*i*_ since variance (SMR)=1/*E*_*i*_. For each area out of *m*, the *p*-value under the null hypothesis *H*_0_:θ=1, Prob(*Y*≥*Y*_obs_∣*H*_0_), can be easily obtained from the exact Poisson distribution.

Bayesian inference requires the specification of appropriate prior distributions on model parameters.

Clayton and Kaldor (1987), assumed a Gamma(*k*,ν) prior distribution for θ_*i*_. The hyperparameters *k* and ν are assumed to be exponentially distributed. In this model, Poisson random variability is filtered out and relative risk estimates are shrunken toward the general mean.

Besag *et al.* (1991) specified a random effect log linear model for the relative risk log(θ_*i*_)=*u*_*i*_+*v*_*i*_. The heterogeneity random term *u*_*i*_ represents an unstructured spatial variability component assumed *a priori* distributed as Normal (0, λ_*u*_) where λ_*u*_ is the precision parameter modeled as Gamma.

The clustering term *v*_*i*_ represents the structured spatial variability component assumed to follow *a priori* an intrinsic conditional autoregressive (ICAR) model. In other words, denoting *S*_*i*_ as the set of the areas adjacent to the *i*-th area, *v*_*i*_∣*v*_*j*∈*Si*_ is assumed distributed as Normal(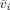, λ_*v*_ *n*_*i*_) where 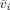 is the mean of the terms of areas adjacent to the *i*-th one (Besag and Kooperberg, 1995) and λ_*v*_ *n*_*i*_ is the precision which is dependent on *n*_*i*_, the number of areas in *S*_*i*_. Through these two random terms the BYM model shrinks the relative risk estimates both toward the local and the general mean.

Apart from the specification of the hyperparameters, both specifications can be viewed as bi-level hierarchical models with the likelihood specified at the first level (Poisson likelihood) and the *a priori* distributions for the parameters of the relative risk function specified at the second.

### 3.3 Hierarchical Bayesian mixture model

If the goal is not estimation, but detection of areas diverging from the reference, we need to complicate the above models by introducing a third level into the hierarchy. This is equivalent to assuming a mixture model for the unknown relative risks θ_*i*_.

The first level of the mixture model is again the Poisson likelihood:


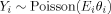


The mixture is introduced at the second level:


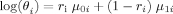


where the logarithm of the relative risk θ_*i*_ is modeled as the mixture of two components: μ_0*i*_, the value of the log relative risk under the null hypothesis, and μ_1*i*_ the corresponding value under the alternative. The *r*_*i*_ indicator denotes the group membership.

Under the null we assume that all the probability mass is concentrated at one point, *i.e.* μ_0*i*_=0, leaving only a Poisson random variability modeled at the first level. Under the alternative extra Poisson variability, which reflects the heterogeneity of relative risk among areas, is modeled according to the two possibilities described above. The first specification is the Poisson-Gamma one, exp(μ_1*i*_) ∼ Gamma(*k*,ν), the second the BYM model, μ_1*i*_=*u*_*i*_+*v*_*i*_. Both the Poisson-Gamma and the convolution (BYM) models require appropriate prior distributions for the hyperparameters, as discussed in the previous section.

The third level of the hierarchy consists in the definition of the prior distribution for the indicator of the unknown true status, *r*_*i*_ that is assumed to be Bernoulli distributed with parameter π_*i*_, which, in turn, is modeled as Beta(*c*,*d*) distribution.

The quantity of interest for each *i*-th area is some appropriate summary measure over the posterior distribution of π_i_ – *i.e.* the posterior classification probability to belong to the set of the null hypothesis (Scott and Berger, 2006).

The *a priori* distribution for π_*i*_ is assumed to be an exchangeable informative Beta(9.7,0.3) with percentiles for π_*i*_ (10% : 0.91; 25% : 0.97; 50% : 0.99) and an expected value (0.97) that are sensible for a prior belief of a small percentage of divergent areas (around 3%).

### 3.4 Sensitivity analysis on hyperprior distribution

Posterior inference on the classification probabilities may be highly dependent on the choice of the prior. We performed a sensitivity analysis changing the parameters of the Beta(*c*,*d*) distribution. We set them to give an expected value of 0.50 (*c*=1,*d*=1) or 0.90 (*c*=9, *d*=1) for the prior belief on the null hypothesis, leaving a reasonable amount of variation (Scott and Berger, 2006, p. 2147).

All the analyses were performed using the WinBugs1.4 software (Lunn *et al.*, 2000). For each model, we ran two independent chains and the convergence of the algorithm was performed following Gelman and Rubin (1992). We discarded the first 100 000 iterations (burn-in) and stored for estimation 50 000 iterations.

## 4 Results

### 4.1 Thresholding *q*-values

**Figure 1:**
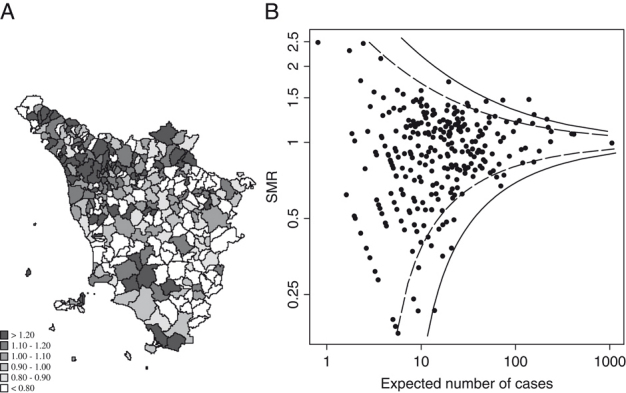
(A) Spatial distribution of SMRs. (B) Funnel plot of SMRs with thresholding lines (dashed: *p*-value<0.05; solid: *q*-value<0.05) (see text). Lung cancer mortality, males. Tuscany, 1995–1999.

The map of SMR for lung cancer among males (Tuscany, Italy) in the period 1995–1999 is shown in Fig. 1(A). The map suggests the presence of high/low risk areas (SMRs range between 0 and 2.5). The reader must remember that we are testing two-sided alternative hypothesis.

In Fig. 1(B), we report the funnel plot (Egger *et al.*, 2001), which is a graph of the effect measure (SMR) against precision (*E*_*i*_ the inverse of SMR variance). This plot is based on the fact that the precision in estimating the risk will increase as the dimension (population) of the area increases. SMR from small areas will spread widely and the pattern will be narrow among larger areas. Under the null, the plot will resemble a symmetrical funnel centered at the reference line at an ordinate value of one. Intervals around the reference line are plotted as dashed lines corresponding to 95% percentiles of the null distribution and intervals adjusted for multiple comparison, using *q*-values thresholded at 5%, are plotted as solid lines. Divergent areas should lie outside the funnel lines (Jones *et al.*, 2008).

**Figure 2:**
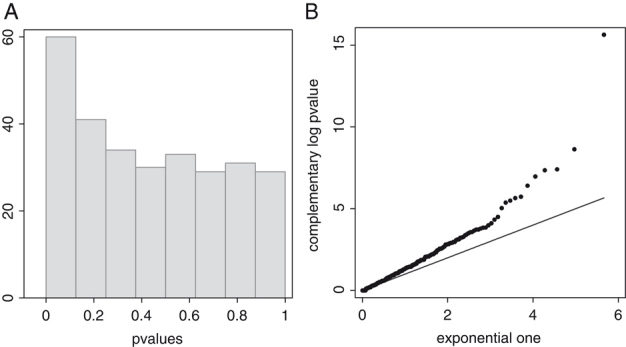
(A) Histogram of empirical *p*-values. (B) Quantile–Quantile plot of complementary log empirical *p*-values *versus* theoretical exponential (1). Lung cancer mortality, males. Tuscany, 1995–1999.

Figure 2(B) shows the empirical distribution of two-sided *p*-values for each *i*-th area obtained from the exact Poisson distribution under the null *H*_0_: RR_*i*_=1. If no areas were to diverge, the *p*-value distribution would be uniform. There is an evidence of a departure from the null hypothesis. Figure 2(B) shows the quantile–quantile plot of the complementary log transformation of empirical *p*-values against the theoretical exponential(1) distribution under the null. The plot highlights some outlying observations.

Over the 287 municipalities 32 had *p*-values<0.05 (see the dashed funnel line in Fig. 1(B)). Applying Bonferroni's correction (with probability of type I error set at 5%) only one area is selected as divergent from the null value of one. Using Storey's *q*-value 11, 6, and 4 areas are selected thresholding at 20%; 10 and 5% respectively (see the solid funnel line in Fig. 1(B)). Storey's estimator of the proportion of true null gives 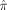
=97%.

### 4.2 Tri-level hierarchical Bayesian models

**Figure 3:**
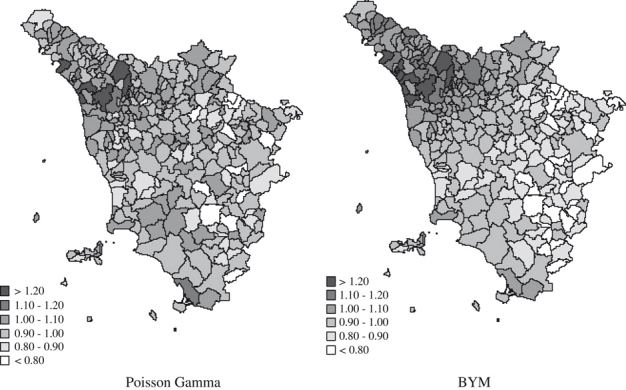
Smoothed relative risk estimates from the tri-level Poisson-Gamma and tri-level BYM models (see text). Lung cancer mortality, males. Tuscany, 1995–1999.

Figure 3 reports the maps of estimated Relative Risks by the proposed tri-level Poisson-Gamma and BYM models. The shrinking effect of the Bayesian estimators is evident when comparing it to the SMR map (Fig. 1(A)). BYM estimates are spatially smoothed and since high risk areas are clustered in the north-west part of the region, while the low risk areas are located in the south-east, the degree of shrinkage toward the general mean is less prominent. Small areas with few expected counts and extreme SMR are regressed to the mean. Since we used indirect standardization with Tuscan reference rates, the observed mean (0.97) was close to the null RR of one. Generally speaking, lung cancer risk among males showed a very strong spatial structure (see Biggeri *et al.*, 2009).

**Figure 4:**
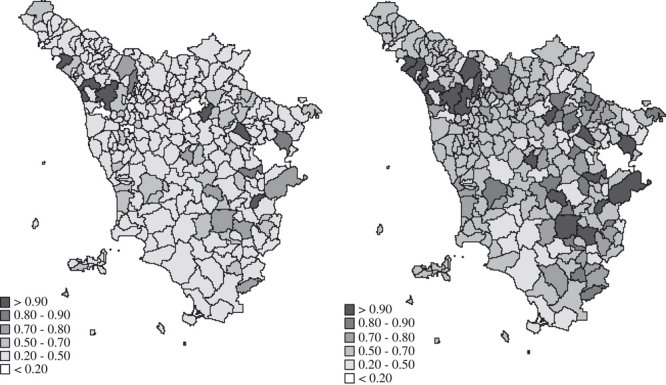
Posterior inclusion probabilities Prob(*r*_*i*_=0 | Y=Y_*i*_; **Y**) under the tri-level Poisson-Gamma and the tri-level BYM models (see text). Lung cancer mortality, males. Tuscany, 1995–1999.

Figure 4 shows the posterior inclusion probabilities (*i.e.* the complement to the posterior classification probabilities) from the proposed tri-level hierarchical Bayesian models. Posterior inclusion probabilities adjust for multiple comparisons. Thresholding at 90%, seven areas under the Poisson-Gamma model and nineteen areas under the BYM model were detected as divergent.

### 4.3 Model-based inclusion probabilities *versus q*-values

**Figure 5:**
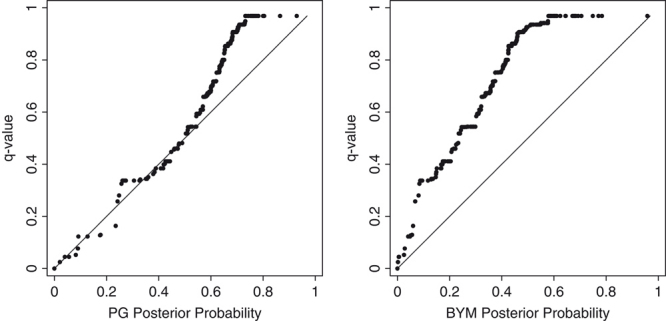
Quantile–Quantile plot of *q*-values against posterior classification probabilities (left PG: tri-level Poisson-Gamma model; right BYM: tri-level BYM model). Lung cancer mortality, males. Tuscany, 1995–1999.

We compared the results from the tri-level hierarchical Bayesian models with those obtained thresholding *q*-values. In Table 1, we classified areas as divergent by cross-tabulating them with posterior classification probabilities under the tri-level hierarchical Bayesian models and by *q*-value. The cut-off level was set at 20%. The proportion of specific agreement *p*_*s*_ (Fleiss *et al.*, 2003) was 86% between tri-level Poisson-Gamma and *q*-value while only 43% between tri-level BYM and *q*-value. Disagreement between tri-level Poisson-Gamma and *q*-value was small and symmetrical. Instead, the spatially structured tri-level hierarchical Bayesian model “discovered” 29 divergent areas that were not detected by the *q*-value. Figure 5 reports the quantile-quantile plots of ordered *q*-values against ordered posterior classification probabilities. If the two criteria produce the same ranking, the areas will lie on the bisector. Ordering on the tri-level Poisson-Gamma posterior classification probabilities is slightly more conservative than using the *q*-value. The tri-level BYM behaves very differently and the selected divergent areas are spatially contiguous (Fig. 4 on the right).

**Table 1:** Number of municipalities identified at altered risk by the posterior classification probabilities under the tri-level Poisson-Gamma and convolution BYM models *versus q*-value. The cut-off level is set to 20%. Lung cancer mortality, males. Tuscany, 1995–1999

Poisson-Gamma posteriorclassification probability	*q*-Value		BYM posterior classification probability	*q*-Value	
			
	No (>0.20)	Yes (≤0.20)		No (>0.20)	Yes (≤0.20)
No (>0.20)	275	2	No (>0.20)	247	0
Yes (≤0.20)	1	9	Yes (≤0.20)	29	11

### 4.4 Sensitivity analysis

Finally, we performed a sensitivity analysis changing the parameters of the Beta hyperprior distribution. First, we specified a Beta(1,1) for π, the probability of the null. This naïf choice, centered at 0.50, produced very unrealistic results. Indeed, as noted by Scott and Berger (2006), the researcher often has strong information about π and the information is that π is close to one. In our analysis we specified a Beta distribution on π with mean value=0.97. We alternatively specified a Beta distribution in order to give an expected value of 0.90 for the prior belief on the null hypothesis, leaving a reasonable amount of variation (first decile 0.77; first quartile 0.86; median 0.93; third quartile 0.97; last decile 0.99).

**Figure 6:**
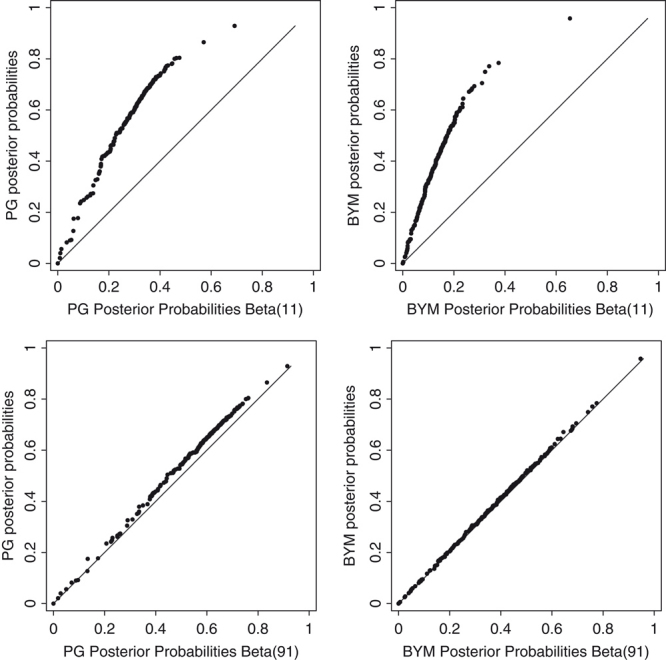
Sensitivity Analysis. Quantile–Quantile plot of posterior classification probabilities (left PG: tri-level Poisson-Gamma model; right BYM: tri-level BYM model) against posterior classification probabilities obtained specifying a Beta(1,1) (upper) or a Beta(9,1) (lower) hyperprior. Lung cancer mortality, males. Tuscany, 1995–1999.

Figure 6 reports the results of the sensitivity analysis. On top, the quantile–quantile plot of posterior classification probabilities under the Beta(9.7,0.3) hyperprior *versus* those obtained under the uniform hyperprior and on the bottom *versus* those obtained under the more spread Beta(9,1) hyperprior.

The uniform hyperprior is inappropriate, as expected. The choice of Beta(9.7,0.3) is robust to moderate change in prior belief.

## 5 Conclusion and discussion

As of yet, no work has been done on FDR and disease mapping. Our work on this topic started in 2006 (Catelan *et al.* 2006) and was motivated by an environmental epidemiological investigation on 18 high-risk areas and more than 30 disease codes. It is not surprising that until now the problem of multiple testing was not considered in disease mapping, because Bayesian approaches to smooth relative risk estimates may be misinterpreted as a solution to the problem. Bayesian estimators are superior to maximum likelihood ones when considering the whole set of estimates (earlier work was empirical Bayesian, see Clayton and Kaldor, 1987; Efron and Morris, 1973). Other epidemiological applications on long lists of relative risks appeared in Greenland and Robins (1991) and Carpenter *et al.* (1997).

However, estimation is a different task from testing. Indeed the inferential goals may be different. With regard to estimating relative risks, their distribution, their ranks, Sheen and Louis (1998) showed that there is no one simple best procedure. Moreover, paraphrasing Müller *et al.* (2007), testing multiple hypotheses requires, first that the probability model include a positive prior probability of the null for each observation, second that the model includes hyperparameters for the null prior.

A full Bayesian model that adjusts for multiple testing consists of a tri-level hierarchical Bayesian model that allows one to estimate the posterior probability to belong to the set of the null or alternative hypotheses. The advantage of using a model-based approach over a simpler frequentist one is twofold. If the model is well specified, there is a gain in power. In our application, the tri-level spatially structured hierarchical Bayesian model detected many more areas as divergent, leaving the impression of a greater power for such specification. But the reader must remember that in the real application, we have no way of telling if an area called divergent at a given threshold is in fact truly divergent. The second advantage of the Bayesian approach is that it easily allows one to perform sensitivity analysis on model assumptions (see for example Briggs *et al.*, 2006) and, in the multiple testing framework, on prior belief of the null (Westfall *et al.*, 1997). Bayesian modeling will also combine risk estimation and multiple testing. Connection of posterior classification probabilities and *p*-values is discussed in the Bayesian literature (Casella and Berger, 1987; Bayarri and Berger, 2000).

A related work to our hierarchical modeling is found in Jones *et al.* (2008) and Ohlssen *et al.* (2007). Different from us, they considered one-sided tests to avoid confusion when flagging health-care providers as good or bad. Their approach is aimed to build prediction limits around the funnel plot analogous to what is showed in Fig. 1(B). In Ohlssen *et al.* (2007), they developed a hierarchical Bayesian model for the null hypothesis aiming to get cross-validation predictive *p*-values. Interestingly, they distinguished between (i) estimating effects within a random effect model (ii) testing a simple null model using *p*-values and (iii) estimating posterior probability of coming from the null within a mixture model. This last point is what we addressed in the present article.

A further point regards the use of a transformation of observed/expected ratios (Grigg *et al.*, 2009). However, we developed a full probabilistic model that did not rely on asymptotics. Some of these details can be found in a simulation study by Catelan and Biggeri (2010), where, in the usual disease mapping framework and specifying a Poisson likelihood, the assumptions underlying the Bayesian interpretation of the *q*-value were checked.

**Figure 7:**
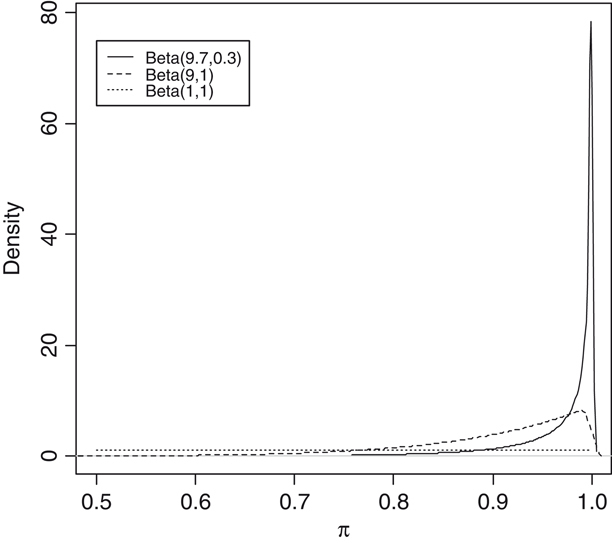
Hyperpriors for π (long dashed: uniform; dashed: Beta 9,1; solid: Beta 9.7, 0.3). Lung cancer mortality, males. Tuscany, 1995–1999.

There is a strong sensitivity to hyperprior choice for π. We rely on subject-specific information to derive informative distributions for π, as shown in the sensitivity analysis. We conclude that the naïf choice of a uniform distribution for π is strongly expected to be in error (see Fig. 7).

Note that we adopted the strategy to let the data itself choose π, but we specified an appropriate informative hyperprior. Storey (2003) and also Efron (2005) applied an empirical Bayesian approach to estimate π. Concerns were raised about such an approach in applications not related to omics (Jones *et al.* 2008). These authors noted that the amount of information about π in the data could be scant whenever the number of tests is not large enough (>one thousand). At least in the disease-mapping context where the number of tests is between one hundred and one thousand, our sensitivity analysis shows that eliciting appropriate informative hyperprior inference on π is important.

This point deserves further explanation. The Benjamini–Hochberg procedure is robust under a variety of conditions. Storey's *q*-value procedures have been shown to be anticonservative when the number of tests is not high, there is lack of independence, and the proportion of true null approaches one (Benjamini *et al.*, 2006; Dudoit *et al.*, 2008). In our application both procedures gave identical results (eleven areas thresholded at level 20%). When specifying a Beta(*c*,*d*) hyperprior with expected value of 0.97 for the probability of the null, the tri-level Bayesian hierarchical Poisson-Gamma model gave also almost equal results (ten areas threshold at level 20%). The interpretation of such results is that the implied prior belief of the null in the frequentist procedure is close to that modeled in the tri-level Bayesian hierarchical Poisson-Gamma model.

Bayesian approaches are based on model and prior assumptions. We applied two models (Poisson-Gamma and BYM), with and without spatially structured random terms, which may help the reader to appraise different data summaries with different etiological clues (see Elliott *et al.*, 2000 and Lawson *et al.*, 1999).

We show a Bayesian approach to multiple testing in the disease-mapping context. It cannot be viewed as an antagonist to the classical Benjamini–Hochberg procedure or its extension. On the contrary, given some prior information and assumptions, Bayesian analysis summarizes the empirical information and uncertainty. If you believe in a spatially structured model, then you would prefer the BYM assumptions. A discussion about how to run a simulation study for comparing Bayesian models was in Lawson *et al.* (2000). This large simulation study documented the robustness of the Poisson-Gamma and BYM models. We provided examples in which the BYM model may suffer from the same inaccuracies of other less flexible spatially structured models (Biggeri *et al.*, 2000; 2003). Further work is necessary to extend these results to inclusion probabilities from the proposed tri-level models.

Dependency was modeled by specification of a CAR process in the alternative hypothesis by specifying a BYM model. In our application, this led to identifying a larger number of divergent areas. We should be prudent about interpreting this result. The BYM model may be closer to the true data generating model than the alternative Poisson-Gamma model, but spatially structured models may also be anticonservative even if the BYM has been proved to behave quite well in large simulation studies (Lawson *et al.*, 2000). We do not want to stress this point, because the reader may be led to be overconfident about a given model. We prefer to show the advantage of the Bayesian approach in performing a sensitivity analysis on model assumptions and on prior belief of the null. Future developments should address FDR-adjusted inference and decision analysis.

Inference on relative risks of selected areas after viewing the data is a natural complement to investigations such as these discussed in our article. Benjamini and Yekutieli (2005) considered simultaneous and selective inference. Yekutieli (2009) presents a Bayesian framework for providing inference for selected parameters. Our model is an example of a random effect model, because the relative risk for a given area is treated as a random quantity rather than fixed. The reason is that in this particular application, area relative risk depends on the prevalence profile of risk factors and on the composition of susceptible individuals of the area population. Prevalence and susceptibility do vary and cannot be assumed as fixed because the population of a given area is a dynamic cohort. As stated by Yekutieli (2009), under a random effect model, Bayesian inference is unaffected by selection. However, simultaneous and selective inference in the disease-mapping context is an interesting topic for future developments in our work.

“Finding posterior distributions of parameters is only part of the Bayesian solution. The remainder involves decision analysis: … (this) means considering the ramifications of various decisions explicitly in terms of loss functions” (Berry and Hochberg, 1999). We did not pursue this task in the present article (for a recent contribution see for example Guindani *et al.*, 2009). Here, as for the explorative purposes usually undertaken in disease mapping, we used posterior classification probabilities without any explicit cut-off in the same spirit as Jones *et al.* (2008).

Hierarchical Bayesian models provide a sensible framework to model classification probabilities in the context of disease mapping and broadly speaking, we recommend FDR-like procedures when exploring divergent areas in disease mapping.
